# Stem Cell Based Approaches to Modulate the Matrix Milieu in Vascular Disorders

**DOI:** 10.3389/fcvm.2022.879977

**Published:** 2022-06-15

**Authors:** Sajeesh S, Shataakshi Dahal, Suraj Bastola, Simran Dayal, Jimmy Yau, Anand Ramamurthi

**Affiliations:** Department of Bioengineering, Lehigh University, Bethlehem, PA, United States

**Keywords:** ECM, regenerative repair, cardiovascular, elastin, collagen, exosomes

## Abstract

The extracellular matrix (ECM) represents a complex and dynamic framework for cells, characterized by tissue-specific biophysical, mechanical, and biochemical properties. ECM components in vascular tissues provide structural support to vascular cells and modulate their function through interaction with specific cell-surface receptors. ECM–cell interactions, together with neurotransmitters, cytokines, hormones and mechanical forces imposed by blood flow, modulate the structural organization of the vascular wall. Changes in the ECM microenvironment, as in post-injury degradation or remodeling, lead to both altered tissue function and exacerbation of vascular pathologies. Regeneration and repair of the ECM are thus critical toward reinstating vascular homeostasis. The self-renewal and transdifferentiating potential of stem cells (SCs) into other cell lineages represents a potentially useful approach in regenerative medicine, and SC-based approaches hold great promise in the development of novel therapeutics toward ECM repair. Certain adult SCs, including mesenchymal stem cells (MSCs), possess a broader plasticity and differentiation potential, and thus represent a viable option for SC-based therapeutics. However, there are significant challenges to SC therapies including, but not limited to cell processing and scaleup, quality control, phenotypic integrity in a disease milieu *in vivo*, and inefficient delivery to the site of tissue injury. SC-derived or -inspired strategies as a putative surrogate for conventional cell therapy are thus gaining momentum. In this article, we review current knowledge on the patho-mechanistic roles of ECM components in common vascular disorders and the prospects of developing adult SC based/inspired therapies to modulate the vascular tissue environment and reinstate vessel homeostasis in these disorders.

## Introduction

The structural and functional homeostasis of mammalian organs is maintained by support provided by their connective tissue components comprising of a three-dimensional network of cells and ECM (1, 2). The ECM provides support and anchorage for the parenchymal cells, and regulates cell fate processes including cell survival, proliferation, adhesion, and migration (3). Fibroblasts resident within most connective tissues, are the primary cell types responsible for secreting the interstitial ECM, which further determines tissue architecture, stiffness and flexibility. Neo-assembly and remodeling of ECM structures mostly occur during fetal development and during the organ differentiation stage. In adulthood, ECM remodeling is usually associated with wound healing and matrix regeneration/repair, following an injury stimulus and subsequent inflammation cascade (4). This is also typical of cardiovascular (CV) disease conditions, which involves some degree of ECM degradation and remodeling, resulting in loss of tissue elasticity and fibrotic tissue formation (5). Both these aspects can lead to impaired tissue and organ function (6). Therapeutic strategies aimed at attenuating adverse fibrotic responses and in stimulating biomimetic ECM regeneration and repair in CV disorders are of prime significance. However, this is still at a nascent stage owing to an incomplete understanding of the activation mechanism, regulation, and modulation of ECM regenerative and reparative processes (6, 7). Challenges include the intrinsically poor matrix regenerative capacity of adult vascular tissues, which primarily contain stable (slow renewing/remodeling) cells, and the limited capability of adult vascular cells to synthesize and organize elastic fibers, and their inability to replicate the biocomplexity of developmental elastic matrix assembly (8–12). In this article, we review current knowledge on how ECM changes serve as the etiological basis for vascular disease manifestations and the prospects of developing SC-based ECM regenerative therapies to reverse vascular pathophysiology.

## ECM Composition and Structure in the Vascular Wall

**Figure 1:**
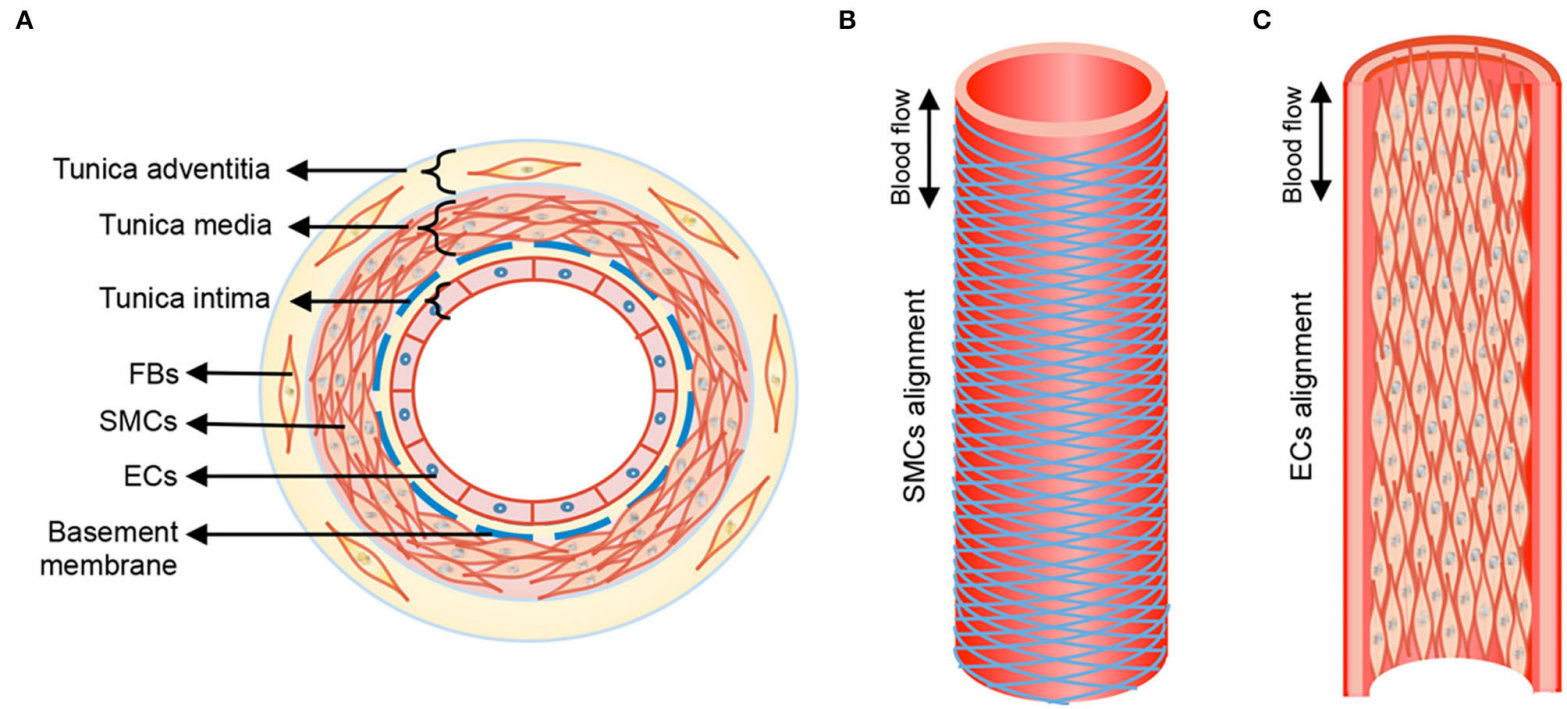
Anatomical structure of blood vessel from transverse **(A)** and longitudinal **(B)**, **(C)** views. Tunica media of blood vessel shows aligned circumference of SMCs following herringbone helical arrangement, and tunica intima form straight cell alignment. FBs, fibroblasts; SMCs, smooth muscle cells; ECs, endothelial cells. Double-headed arrow, blood flow direction. Reprinted from Wang et al. (14), with permission from IOP Publishing Ltd.

The walls of healthy blood vessels exhibit a lamellar structure, with the concentric layers variably containing endothelial cells (EC), smooth muscle cells (SMC), and fibroblasts distributed in a layer-specific ECM milieu (13). Medium-sized muscular arteries and large elastic arteries exhibit 3 distinct tissue layers, namely tunica intima, tunica media, and tunica adventitia (Figure 1). The tunica intima lines the blood vessel lumen and comprises a monolayer of ECs that organize along blood flow and are anchored on a proteoglycan (PG) rich basement membrane that separates these cells from the underlying mesenchymal tissues. ECs regulate thrombosis, fibrinolysis, leukocyte adhesion and extravasation, and also serve to regulate vascular tone through their signaling of SMCs in the underlying medial layer. The middle layer, the tunica media, is separated from the innermost tunica intima layer by internal elastic lamina, a dense layer of concentric elastic fibers generated by intervening SMCs. The tunica media layer accounts for the bulk of the vessel wall thickness in the muscular and elastic arteries and is the chief determinant of mechanical compliance of the wall. The tunica media is composed of circumferentially arranged elastic lamellae, interspersed with SMCs, matrix collagens, microfibrillar glycoproteins, PGs, and amorphous ground substances. In this specific arrangement, the cells and the ECM play major roles in mechano-sensing and in providing force resistance with direct implications to maintenance of vascular wall homeostasis. More broadly, the tunica media is the primary load bearing layer of the vessel at physiologic blood pressures, but it also provides the vessel compliance to accommodate changes to blood flow toward regulating blood pressure. The outermost tunica adventitia, also called the tunica externa, is separated from tunica media by the external elastic lamina. The tunica adventitia is composed of compact (closer to tunica media) and looser (toward outer edge) collagenous ECM, fibroblasts, perivascular nerves, lymphatic vessels, vasa vasorum, and inflammatory cells. However, the exact composition of this layer depends on arterial size and their function. The tunica adventitia serves to prevent vessel over-expansion and rupture at super-physiologic blood pressures and anchor the vessels to the surrounding tissue/organs.

**Figure 2:**
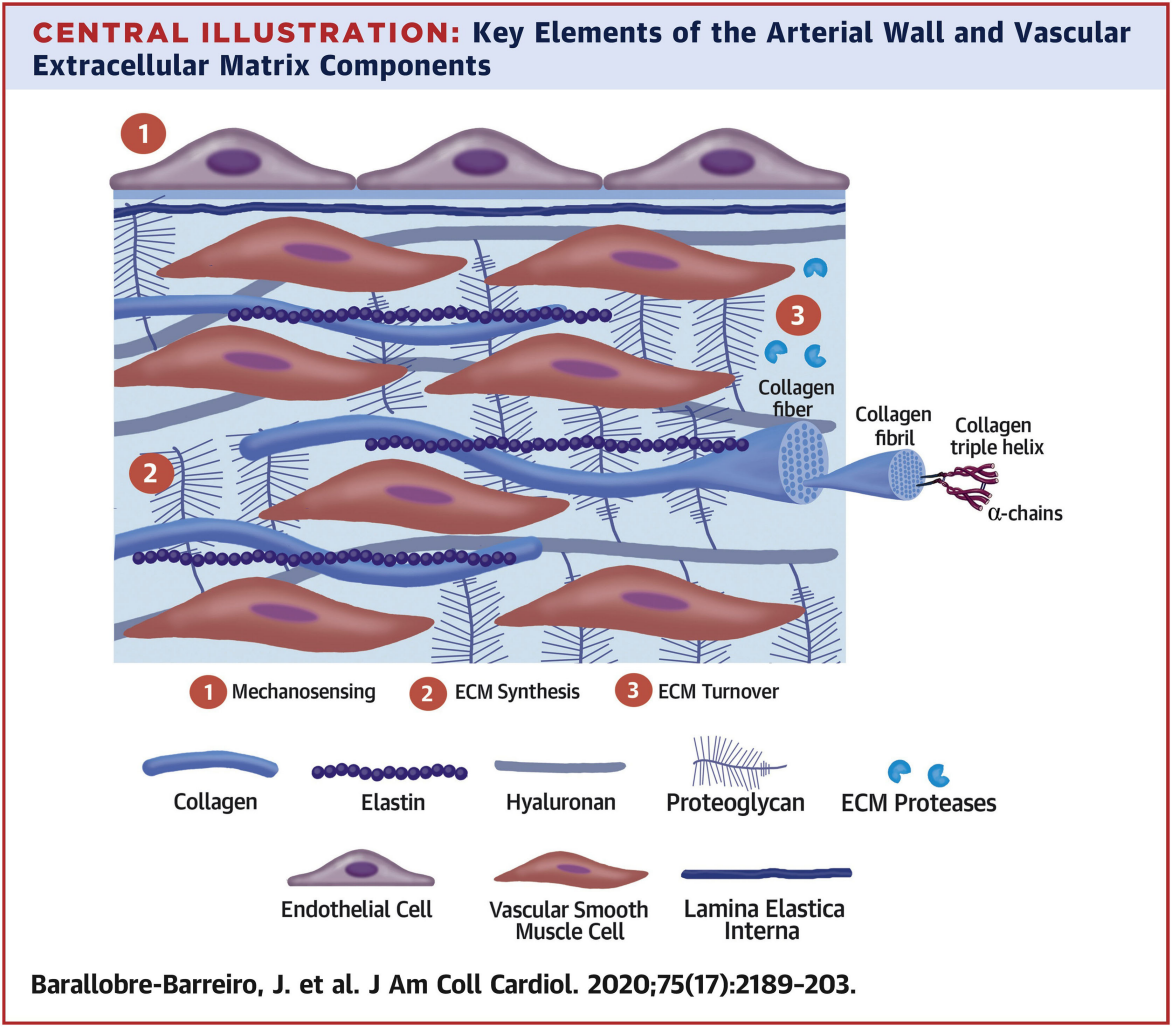
Key elements of the arterial wall and vascular extracellular matrix components. Reprinted from Barallobre-Barreiro et al. (5), with permission from Elsevier.

The ECM secreted by vascular cell types (SMCs, fibroblasts, and less so, ECs) allows the vessel wall to adapt to mechanical forces encountered. The mechanical and viscoelastic properties of the vessel wall (i.e., high resilience, low hysteresis, and non-linear elasticity) are mostly imparted by 3 main structural ECM components: elastic fibers, collagen fibers, and large aggregating PGs (3, 13) (Figure 2).

### Elastin in the Vessel Wall

**Figure 3:**
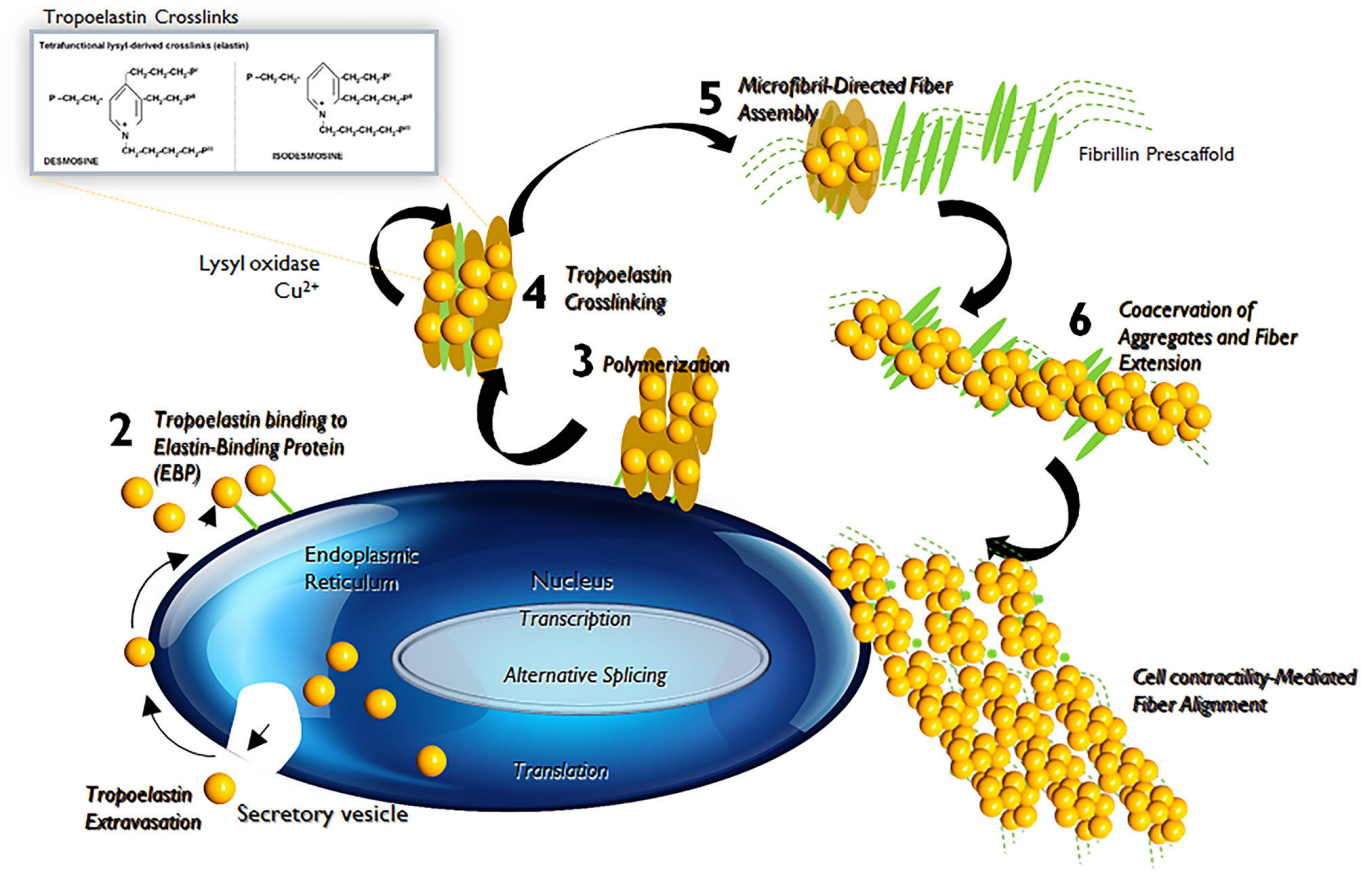
Tropoelastin synthesis, binding with elastin-binding protein (EBP), transport, release of EBP, assembly with fibulins, binding to microfibrils, lysyl oxidase-mediated cross-linking, and final formation of an elastic fiber with microfibrils.

Elastin is the chief protein constituent of elastic fibers, and accounts for ~90% of its composition by mass (15, 16). Elastic fibers provide stretch and recoil properties to soft, pliable tissues of vertebrates, including in blood vessels. Elastin also play a critical role in influencing the phenotype and cell fate processes of interacting cell types, primarily, the SMCs (17). Elastin is formed through the multimerization and subsequent cross-linking of its hydrophobic monomers, called tropoelastins (18) (Figure 3).

In the aorta, elastin is predominantly produced by medial SMCs, though adventitial fibroblasts and ECs also exhibit limited elastogenic capacity. The process of elastic fiber neo-assembly is initiated during fetal development, and this serves as counterbalance to the mechanical forces acting on the forming vascular conduits, to enable their elastic recoil. Repeated stretch and relaxation cycles allow the tropoelastin monomers to polymerize through a process termed as coacervation (19). Tropoelastin isoforms demonstrate a specific pattern of the molecular arrangement, in which hydrophobic regions alternate with hydrophilic cross-linking domains containing lysyl residues (20). The proximity of lysine residues further allows covalent cross-linking, through oxidative deamination facilitated by lysyl oxidase (LOX), to form linkages called desmosines and isodesmosines. This process, directed by arranging hydrophobic and hydrophilic sequences in the tropoelastin molecule determines the intermolecular alignment in the protein and leads to the formation of durable and metabolically inert cross-linked tropoelastin arrays (21, 22). Mature elastic fibers are formed by deposition and aggregation of these crosslinked tropoelastin coacervates on microfibrillar pre-scaffolds which are laid down by cells in the extracellular space (23). These microfibrillar pre-scaffolds are mainly composed of heterogeneous glycoproteins (such as fibrillin-1 and fibrillin-2), microfibril- associated glycoproteins (MAGP1 and MAGP2) and latent transforming growth factor β (TGF-β)-binding proteins. The interaction of these microfibrils with chondroitin sulfate PGs (e.g., versican, biglycan, and decorin) and other proteins present at the elastin–microfibril interface or at the cell surface–elastic fiber interface, known as elastin microfibril interface-located proteins (EMILINs), also serves to play a role in elastogenesis process (24).

Tropoelastin molecules are rich in hydrophobic amino acid residues and are amenable to robust crosslinking. Thus, fully polymerized mature elastin is practically insoluble in aqueous environments, metabolically inert, and extremely resistant to thermal and chemical onslaught (15, 17). For these reasons, elastin has an estimated half-life of about 40 years and under optimal conditions, might be expected to last over the normal human life span. However, defects in vascular elastic fibers or their assembly, either driven by genetic factors and/or disease/injury can compromise the biomechanics and biochemical regulation of vascular cells leading to the adverse remodeling characteristic of several vascular disorders (25).

Deregulation of vascular SMCs impairs their ability to switch between a differentiated phenotype (also referred as “contractile”) and dedifferentiated phenotype (also referred as “synthetic”). Differentiated vascular SMCs exhibit high levels of contractile gene expression and demonstrate low levels of proliferation, migration and ECM synthesis, while the dedifferentiated state is often linked to increased rates of proliferation, migration and production of ECM (26). Upon injury, SMCs acquire a dedifferentiated state to promote vessel repair and cells return to a non-proliferative phenotype once the injury is resolved. Phenotype modulation of vascular SMCs is critical to maintaining vessel wall homeostasis. Several physiological and non-physiological stimuli can deregulate the vascular SMC phenotype switch, contributing to the initiation and progression of several vascular disorders.

Differently, chronic proteolytic breakdown of elastic fiber structures due to localized imbalances between elastin degrading matrix metalloproteases (e.g., MMPs 2, 9) and their inhibitors (tissue inhibitors of MMPs, TIMPs), with increased levels of the former, can also lead to the same adverse remodeling outcomes (27). In the latter situation, the pathological scenario is exacerbated by the generation of elastic fiber breakdown products, elastin derived peptides (EDPs), which contrary to intact elastic fibers, promote a differentiated SMC phenotype characterized by increased cytokine and MMP release, reduced cell contractility and increased proliferation, enhanced intracellular calcium ion uptake, and apoptosis, all of which enhance vascular ECM breakdown *via* positive feedback (28).

### Collagen in the Vessel Wall

Collagen is one among the major structural components of the ECM that provide integrity and stability to the vascular wall (29). Collagens are typically transcribed and secreted by fibroblasts as a precursor, procollagen, which undergoes several post translational modifications and LOX-mediated crosslinking to form triple helical collagen superstructures (30). Collagens with their unique architectural compositions and polypeptide α-chains curled around each other resulting in the formation of triple helix structure, can form intricate ECM networks. Additionally, collagens also contain non-triple-helical domains, which usually serve as the attachment sites for other ECM proteins. Collagens vary by the length of these amino acid repeat motifs, and this permits the formation of supramolecular aggregates that can arrange into varying geometric networks to allow functional diversity. The supramolecular assembly of collagen fiber bundles is further directed through a combination of tissue-specific matrix macromolecules, such as fibronectin and PGs, cell surface integrins, and intracellular forces.

The major collagens in large elastic arteries are the fibrillar collagens (types I, III, V) that provide structural/biomechanical functions. Non-fibrillar collagens (type IV and VI), which form part of the basement membrane, are involved in anchoring and organizing fibrillar collagens and have cytoprotective roles (31). Collagen type I (70–75%) and type III (20–25%) are the prominent subtypes found in human arterial wall, while type V accounts for 1–2% of total collagen content. Fibrillar collagens are enzymatically cross-linked during deposition stage *via* LOX and LOX–like proteins and small leucine-rich proteoglycans (SLRPs) contain collagen-binding domains. This allows the formation of a protein coat on the surface of the fibrils and further protects them from protease degradation (32). The interaction of fibromodulin with collagen cross-linking sites further activates LOX–like proteins and enhances collagen cross-linking process. Thus, collagen plays a key role in maintaining structural integrity of the vessel wall and impaired collagen metabolism has been associated with several CV inflammatory conditions (33).

### Proteoglycans in the Vessel Wall

PGs composed of protein cores that are decorated with glycosaminoglycan (GAG) side chains (34). GAGs are linear, anionic, unbranched polysaccharides made up of repeating disaccharide units and are divided into two groups: sulfated (e.g., chondroitin sulfate, heparan sulfate, and keratan sulfate) and non-sulfated (hyaluronic acid) GAGs. Often, GAG chains are attached to a single protein core and may link at one end to another GAG resulting in the formation of a huge macromolecule. Despite of their small constituting fraction in the normal arterial wall, studies have shown that interaction of GAGs/PGs aggregates with other ECM constituents play a key role in maintaining the bio-mechanical properties of the vessel wall.

PGs are extremely diverse in size, shape and chemistry, and their structure is based on their core proteins, location, and GAG composition. PG content in ECM of normal vascular tissue is low, however this dramatically increases in almost all stages of vascular diseases. PGs accumulate largely in lesions of the vasculature that are prone to disease initiation and are frequently coincident with early phases of atherosclerotic lesion formation through the retention of cholesterol-rich lipoproteins (35). PGs are also involved in ECM related metabolic processes and crosstalk with other inflammatory cells that extravasate to the subendothelial regions (36).

## ECM Degradation and Remodeling

The vascular ECM is critical to maintain vascular wall integrity, to impart tensile strength, viscoelasticity, elastic recoil and compressibility to the vessel wall. The ECM also plays a key role in regulating the phenotype and cellular fate of vascular cell types, through the distinct properties of its different constituents (4, 5, 7). ECM degradation and remodeling are hallmarks of several vascular diseases, including hypertension and arterial stiffness, atherosclerosis (AS), and aortic aneurysms (AA; Table 1).

**Table 1:** List of major vascular disorders caused by ECM degradation and remodeling.

**Disease**	**Etiology**	**Features**	**References**
Hypertension and arterial stiffening (Arteriosclerosis)	Acquired	Altered collagen metabolism, higher MMP expression, reduced amount of elastin or compromised fiber assembly, altered ECM composition, generation of elastin derived peptide etc. causes arterial stiffening and leads to hypertension and other complications.	(37–41)
Atherosclerosis	Acquired	Chronic inflammation, infiltration of lipids and macrophages, accumulation of PGs, degradation ECM components in the aortic wall leads to formation of AS plaque. Leading cause for myocardial infraction and stroke.	(42–47)
Abdominal Aortic Aneurysm	Acquired	Infiltration of inflammatory cells, chronic over expression of MMPs (MMP 2 and 9) cause medial SMC apoptosis and ECM degradation, destruction and fragmentation of elastic fibers in the arterial wall. Compensatory collagen deposition mechanically stabilizes weakened aortic wall, but eventually leads to a fatal hemodynamic stress-induced wall rupture.	(48–58)
Thoracic Aortic Aneurysm	Genetic	Abnormalities in elastin-associated microfibrils caused dysfunctional FBN1 gene and increase tissue level of TGF-β, leads to breakdown of elastin.	(59–64)

### Arterial Stiffness and Hypertension

Arterial stiffness, also referred as arteriosclerosis, is one of the earliest clinically detectable manifestations of impaired vascular ECM remodeling (37). Altered collagen metabolism is often considered as the major contributor to the pathogenesis of arterial stiffness (38). Remodeling of the arterial wall is mainly driven by collagenases or MMPs and stiffened vessels usually show increased deposition of collagen and higher expression of MMPs. These vessels also have fragmented and reduced elastin content, disorganized endothelium, and infiltrated macrophages and other mononuclear cells (37). Calcium deposition within the arterial wall increases with increased elastic matrix breakdown and generation of bioactive EDPs increases with growing age, contributes to wall stiffening (39). The proportion of senescent cells within the vasculature also increases with age and this further exacerbates the state of chronic inflammation. Thus, the combination of chronic inflammatory events with reduced cross-linked elastin content, accompanied by the elevated levels of activated MMPs and other proteases can seriously compromise the integrity of the elastin-collagen networks and the basement membranes (39). The compensatory increase in collagen production at this stage, directed toward restoring the integrity of vessel wall, often results in the formation of poorly organized and highly cross-linked (stiffer) collagens.

Arterial stiffness increases with advancing age and is associated with a higher risk of developing hypertension (38). However, vascular stiffening and hypertension appear to be bidirectionally associated (40). High blood pressure may induce vascular damage and accelerate the artery stiffening process, while aortic stiffening increases pressure pulsatility and affects systolic blood pressure (40). The chronological relationships between vascular stiffness and blood pressure remain ambiguous, whether with the cause-effect relationship between vascular stiffness and hypertension or vice versa yet to be completely elucidated (41). Nevertheless, the clinical combination of hypertension and arterial stiffness marks a major step toward the development of serious CV complications.

### Atherosclerosis

AS is a common cause of coronary artery disease and stroke and is a leading cause of morbidity and mortality worldwide (42). AS refers mainly to the lipid deposition that occurs within lesions around the tunica intima, followed by the migration and proliferation of SMCs from the tunica media layer. These events subsequently lead to the formation of atherogenic lesions or fibroid plaque lesions. Even though the exact reason for AS plaque formation is currently unknown, experimental and clinical evidence suggest that AS is a chronic inflammatory disease (42–45). Hypercholesterolaemia is considered one of the main causes of AS formation; an increase in plasma cholesterol levels alter arterial endothelial permeability and allows the accumulation of lipids, especially low-density lipoprotein cholesterol (LDL-C) particles, to the arterial wall (43). At this stage, blood leukocytes (primarily lymphocytes and monocytes) migrate and adhere to the damaged ECs, further express vascular adhesion molecule-1 (VCAM-1) and selectins (43). Once attached, these cells produce inflammatory factors, promoting monocytes differentiation into macrophages and leads to the formation of foam cells by engulfing oxidized LDL particles. Uptake of lipid particles makes macrophages less mobile and promotes the accumulation of these lipid-laden cells in the intimal layer (43, 44). These foam cells maintain their metabolic activity and further releases a variety of cytokines and other inflammatory mediators (45). Together with damaged ECs, these foam cells initiate an inflammatory cascade to promote the proliferation of vascular SMCs toward a synthetic pro-atherogenic phenotype and supports their trans-endothelium migration. Under normal physiological conditions moderate proliferation of SMCs is associated with healthy vascular wall repair, however excessive activation is often associated with senescence, secondary necrosis, ECM formation and fibrosis. Thus, SMC proliferation and migration play a critical role in the arterial remodeling associated with AS (46).

Recent studies have indicated the role of PGs and GAGs in orchestrating AS progression (36). According to the response-to-retention hypothesis, the key initiating early event in AS is binding of cholesterol-containing lipoprotein particles to newly accumulated intimal PGs, in particular versican and biglycan, results in the gradual lipid deposition in the subintimal space (47). Accumulation of versican is seen in early stages of AS and this predisposes the vessel to lipoprotein retention (65). Peripheral literature also suggests that versican build up is detrimental to tropoelastin production and fiber assembly, and their accumulation occurs mainly in response to aging, hypertension, and other vessel injury (66). Thus, pro-atherogenic stimuli affecting versican expression or its GAG composition could contribute to plaque formation and alter ECM state to in turn trigger changes to phenotypes of contacting vascular cells (67).

In AS, ECM remodeling is considered a critical step in development and progression of the condition, the dynamic interactions between the multitude of cell types and molecular mechanisms involved in regulating the matrix remodeling process are still not fully understood (46). In AS, infiltrating leukocytes are known to release certain proteases that degrade the ECM, and lead to changes in the tissue milieu allowing SMC proliferation and plaque growth (68). AS plaques can alternate between stable and susceptible states depending on this internal environment. Lesions with large lipid core and thin fibrous cap, with higher macrophage infiltration, are usually unstable and prone to rupturing (69–71). Once formed, SMCs maintain stability of AS plaque through secretion of interstitial collagen and MMPs secreted by the activated macrophages can effectively degrade this interstitial collagen and other ECM proteins (69). This makes the AS plaques vulnerable for rupture leading to arterial occlusion, resulting in the formation of major CV complications, including myocardial infraction (MI) and stroke (71). Collectively, these indications clearly suggest that ECM and related proteins play a critical role in the formation and progression of AS disease.

### Aortic Aneurysms

Aortic aneurysms involve progressive enlargement or bulging of the aorta that has the propensity to expand and rupture (48). The most common forms of aortic aneurysms are abdominal aortic aneurysms (AAA) and thoracic aortic aneurysms (TAAs). AAAs, the more prevalent form of aortic aneurysm, are typically associated with advanced age and AS, with risk factors including hyper-cholesterolaemia, hypertension and/or diabetes (49). On the other hand, TAAs occur in all age groups and are more closely associated with hereditary factors and do not necessarily show close association with major identified risk factors for CV disease (e.g., hyper-cholesterolaemia, hypertension, diabetes etc.) (59).

### Abdominal Aortic Aneurysms

AAAs are localized, focal dilatations of the abdominal aorta, the segment between the renal bifurcation and iliac bifurcation (48). An aortal expansion qualifies as an AAA if the diameter of the abdominal aortal segment increases to at least one and half times the original diameter of the healthy vessel (48). The etiology of AAAs is multifactorial (e.g., chronic hypertension, AS, smoking, or vasculitis), but in general, the pathophysiology involves breakdown and loss of aortal wall ECM structures resulting in gradual wall thinning, weakening, and ultimate rupture (50). Histology of clinical AAA tissues show infiltration of leukocytes, degradation of ECM structures, particularly the elastic matrix, and the depletion and apoptosis of medial SMCs to be the major pathological hallmarks of AAAs (51, 52). Although the mechanisms of the disease formation is unknown, studies on animal models of this disease have indicated localized injury stimulus-incited infiltration of inflammatory and immune cells (macrophages, neutrophils, mast cells, T and B lymphocytes) into the abdominal aorta wall (53). These infiltering cells chronically overexpress cytokines and ECM proteases (mostly MMP-2 and 9) and cause medial SMC apoptosis and ECM degradation with gradual loss of aortic wall integrity. Thus, the destructive pathological remodeling of aorta in AAA involves four interrelated factors, namely (a) chronic inflammation of the outer wall of aorta along with neovascularization and upregulation of proinflammatory cytokines, (b) hyper-production and dysregulation of matrix degrading enzymes, (c) progressive destruction of elastin to generate bioactive EDPs, and of collagen and (d) apoptosis of medial SMCs incited by the EDPs (54, 55).

ECM degradation in AAAs occurs due to a chronic imbalance between elastolytic proteases (MMP2 and MMP9), generated by pro-inflammatory macrophages infiltrating the injured aorta wall, and their natural inhibitors, the TIMPs. SMCs in the healthy adult vascular wall are of contractile phenotype and are intrinsically deficient in their capacity for elastin synthesis and have impaired ability to assemble mature elastic fibers (56). Further, diseased SMCs are even less amenable to new elastic fiber assembly and moreover, the degraded state of pre-existing fibers which are critical to the new fiber assembly process is a serious impediment to any prospects of new elastic fiber generation (57).

The decrease in total aortal elastin content in the vessel wall due to chronically upregulated proteolytic activity is compensated by the cellular deposition of collagen in response to enhanced flow-induced stresses encountered by SMCs in the thinning vessel wall. While adult cells, more so, diseased vascular cells, are intrinsically deficient in their ability to generate elastic matrix, and have impaired ability to assemble mature elastic fibers, vascular SMCs and fibroblasts readily and exuberantly regenerate collagen (58). This compensatory collagen deposition mechanically stabilizes the weakened aorta wall for short term thereby delaying AAA rupture. However, the continued degradation of elastin and generated EDPs incite SMC apoptosis, a positive stimulus for proteolytic breakdown of collagen matrix. The resulting imbalance between collagen synthesis and breakdown ultimately leads to fatal hemodynamic stress-induced wall rupture. Thus, early intervention in this pathophysiologic sequence of events to restore elastin homeostasis in the AAA wall could hold potential to slow or regress AAA growth to rupture (58, 72).

### Thoracic Aortic Aneurysms and Dissections

Thoracic aneurysms are classified as aortic root or ascending aortic aneurysms (60). The common characteristic of TAADs involves cystic medial degeneration, which manifests as degenerated elastic fibers, disorganized collagen fibers, and accumulated PGs, besides a contractile to activated phenotype switch of medial SMCs, and later apoptosis of these cells (60). In healthy vessels, vascular SMCs maintain ECM homeostasis by maintaining a perfect balance between secreted proteases (MMPs) and their inhibitors (TIMPs). However, in the TAAD wall, MMPs are chronically overexpressed, causing accelerated ECM degradation. The bioavailability of cytokines and growth factors involved in signaling pathways that regulate ECM homeostasis, which are sequestered in the ECM in its latent form (e.g., TGF-β), are also enhanced (61). These pathological events progressively lead to the weakening of the aortic wall, and further reduce their ability to endure the biomechanical forces imposed by pulsatile blood flow and blood pressure.

TAADs typically demonstrate a classical Mendelian inheritance with high or complete penetrance. This possibly suggest the contribution of a single gene defect in the progression of TAADs. TAADs can be further classified into syndromic presentations that demonstrate characteristics of a systemic connective tissue disorder [e.g., Marfan syndrome (MFS), Loeys–Dietz syndrome (LDS)], and non-syndromic presentations (e.g., isolated familial TAAD syndrome). MFS is a typical heritable autosomal-dominant disorder caused by mutations in a structural glycoprotein fibrillin 1, which is a major component of the microfibrils critical to elastic fiber assembly (62). These mutations result in a decrease in the amount of elastin in the aorta wall and lead to the disorganization of elastic fibers. Moreover, dysfunctional fibrillin-1 microfibrils impair the sequestration of TGF-β1 in the ECM and this causes an increased bioavailability of TGF-β1. As a consequence, the aorta exhibits progressively lower ability to stretch and recoil and increased wall stiffness and dilatation (63). Presenting similar manifestations as MFS, LDS is linked to heterozygous mutations of the TGF-β receptors I & II (TGF-βR1 and TGF-βR2), suggesting non-involvement of TGF-β1 early signaling (64). Cystic medial degeneration in absence of overt connective-tissue disorders, now referred as familial TAA syndrome, is caused mostly through defect in ACTA2 gene which encodes smooth muscle α2 actin. Mutation in ACTA2 leads to vascular SMCs with disorganized and aggregated actin filaments, and subsequently impairs their cellular adaptation to local mechanical stress in the aortic wall (60).

## Stem Cells for Vascular Tissue Repair

SCs represent a unique population of undifferentiated cells which are capable of extensively differentiating into different tissue and cell types (73). They are characterized largely by their ability to self-renew, clonality and potency, but this can vary depending on the type of SCs (74). SCs are increasingly studied in the context of use as model systems to understand cellular mechanism involved in the disease progression, and for their utility in the treatment of a spectrum of disease conditions such as CV disorders, diabetes mellitus, chronic myeloid leukemia, cirrhosis, pulmonary fibrosis, inflammatory bowel disease and disorders of the nervous system (73, 75–77).

### Types of Stem Cells

SCs are classified broadly based on origin as embryonic stem cells (ESCs), induced pluripotent stem cells (iPSCs) and adult SCs (74). ESCs are pluripotent in nature and their unlimited proliferation potential makes them excellent choice for regenerative therapy. However, their use raises several ethical issues and direct use of undifferentiated ESCs for tissue transplant pose potential tumorigenicity concerns. iPSCs are SCs generated by genetically reprogramming adult somatic cells to form an “ESC-like state” and they share similar characteristics with ESCs in terms of morphology, proliferation, and their ability to differentiate into cells of all three germ layers in culture (78). Use of iPSCs raises minimal ethical concerns and change be excellent choice for tissue regeneration and repair.

Adult SCs are the common cell type for therapy and tissue repair, specifically for CV disorders, largely due to their anti-inflammatory properties as well as tissue repair capabilities (75). Transplantation of adult SCs has shown to restore damaged organs *in vivo* and initiate revascularization of the ischemic cardiac tissue through differentiation and generation of new specialized cells (75, 77). Moreover, use of adult SCs has been associated with minimal ethical concerns. The advantage of adult SCs in the clinics and evidence supporting their therapeutic effectiveness in regeneration and repair, makes them suitable choice for therapeutic applications. MSCs are widely used adult SC type for regenerative therapeutics (76, 77). MSCs are multipotent adult cells with the potential to differentiate into multiple cell types like osteoblasts, chondrocytes, myocytes, adipocytes, etc. (76). Mesenchyme refers to the embryonic loose connective tissue derived from the mesoderm and develops into hematopoietic and connective tissue. MSCs are mostly isolated from the bone-marrow tissues but can be obtained from other sources such as umbilical cord, endometrial polyps, menses blood and adipose tissues (75–77).

## Stem Cell Therapy for Major Vascular Disorders

### Stem Cell Treatments for Atherosclerosis

SCs, specifically MSCs, have been shown to impart strong anti-inflammatory and immunomodulatory responses against pathological events centric to vascular diseases, such as AS (79, 80). Considering AS as a chronic inflammatory disease linked to dysregulation of the immune system, an MSC-based therapy could exert protective effects on the progression of AS (81). A growing body of evidence suggests that transplanted MSC can modulate cytokine and chemokine secretion, reduce endothelial dysfunction, promote regulatory T cell function, decrease dyslipidemia, and stabilize vulnerable plaques in the AS region (82–84). Table 2 summarizes key recent animal studies that investigated therapeutic benefits of MSCs in animal models of AS.

**Table 2:** Animal studies involving use MSCs for treatment of Atherosclerosis.

**AS model**	**MSC source**	**Major outcomes with MSC treatment**	**References**
Ldlr–/– mice, with high fat diet	BM-MSC (mice)	Reduction in effector T cells, circulating monocytes and serum CCL2 levels. Reduced dyslipidemia in mice.	(82)
ApoE^−/−^ mice	Skin-derived MSCs (mice)	S-MSCs capable of migrating to AS plaque and selectively taking up residence near macrophages. Reduced the release of the TNF-α and increased the expression IL10 in the plaque region.	(83)
ApoE^−/−^ mice	BM-MSC (ApoE-KO mice)	Reduced the size of AS plaques 3 months after treatment. Atheroprotective role by enhancing the number and function of Tregs and inhibiting the formation of macrophage foam cells.	(85)
New Zealand rabbits, LN2 frostbite AS model	BM-MSC (rabbit)	Downregulation of plasminogen activator inhibitor 1 (PAI-1), and MMP-9 after 4 weeks of MSC transplantation. Serum hs-CRP, TNF-α and IL-6 were significantly down-regulated, whereas IL-10 was significantly up-regulated. Formation of abundant collagen fibers at the plaque rupture areas.	(86)
Ldlr–/– mice, with high fat diet	CM from AD-MSC	Suppression of macrophages accumulation, downregulation of MAPKs and NF-kβ leading to M1/M2 polarization, downregulation of CAM and JNK phosphorylation with CM treatment.	(87)
Japanese big-ear white rabbits with high fat diet	UC-MSC	Downregulated apoptosis and proliferation of arterial cells. EC density increased in treated group. Reduced levels of TNF-α and IL6.	(88)

Undifferentiated MSCs have the capacity to modulate and reduce inflammation and can alter immune cell components within AS plaques to alleviate inflammation (83). The protective properties of MSCs on AS lesions have been largely attributed to the secretion of various anti-inflammatory mediators (80). Transplantation of BM-MSCs into AS lesions in various animal models has been found to result in increased secretion of anti-inflammatory cytokines, including Interleukin-10 (IL-10) and TGF-β1, while the production of pro-inflammatory cytokines, such as IL-1β, IL-6, and Tumor Necrosis Factor (TNF-α), was reduced (Table 2). MSC treatment enhanced IL-10 secretion, which further promoted anti-atherogenic effects primarily by inhibiting macrophage activation, MMPs, and pro-inflammatory cytokines (84). TGF-β1 secretion by MSCs also caused the induction of CD4^+^CD25^+^Foxp3^+^ regulatory T (Treg) cells from non-regulatory cells and also suppressed the proliferation of NK cells in Apolipoprotein E (^−/−^) (ApoE^−/−^) mice AS model (85). The increased ratio of Tregs over CD4^+^ T cells in turn promoted macrophage differentiation toward the M2 phenotype, and thereby reduced monocyte infiltration and inflammatory response in the plaque region (85). These results clearly indicate the beneficial anti-AS role played by MSCs, *via* (a) reducing pro-inflammatory responses, (b) promoting anti-inflammatory environment, and (c) mitigating monocyte recruitment to the lesion site.

Endothelial dysfunction is one of the earliest events in AS initiation (89). MSCs have been shown to successfully restore endothelial function by halting atherogenesis (90). Expression of the vasodilator molecule endothelial nitric oxide synthase (eNOS) locally in the injured vessel wall is responsible for the production of vascular nitric oxide (NO), and this often is associated with AS progression. MSC transplantation has been reported to attenuate AS by improving EC function *via* the Akt/eNOS pathway by upregulating IL8 and Macrophage Inflammatory Protein (MIP)-2 in ApoE^−/−^ mice (90). Since IL-8 is an important pro-atherogenic cytokine involved in the early stages of plaque formation, this study implies the importance of proper timing of cell therapy to be able to prevent growth of an early plaque.

Hyperlipidemia is another well-established risk factor for AS formation and studies have revealed that MSC treatment can reduce lipid levels in various hyperlipidemic animal models (91, 92). MSCs seems to have indirect effect on cholesterol metabolism through immune modulation, however the exact mechanism remains vague. MSC treatment has been effective in reducing plasma cholesterol level in low-density lipoprotein-receptor knockout mice (LDLR^−/−^ mice) due to reduction in very-low-density-lipoproteins (VLDLs) levels (92). This was attributed mainly due to the reduced activation of Kupffer cells, which express mediators promoting VLDL secretion. MSC treatment was effective in reducing SREBP-1c expression, a transcription factor involved in fatty acid biosynthesis, and an increase in PPAR-α expression, a transcription factor modulating fatty acid β-oxidation (93). Overall, this study suggests that MSC administration lowers serum lipid levels and might subsequently reduce lipid accumulation in plaques. MSC-mediated anti-inflammatory signaling appears to be the mechanism behind lipid reduction *via* altered lipid metabolism, however further studies are required to validate these observations.

MSCs have the potential to treat advanced AS plaque lesions through the regeneration of the inner endothelial lining and collagen fiber formation in the vessel wall (94, 95). MSC transplantation stabilized vulnerable plaques in AS rabbit model through immune modulation, by reducing the expression of pro-inflammatory cytokine TNF-α and IL-6 and by increasing IL-10 expression (86). Moreover, the expression of MMPs (MMP-1, MMP-2, and MMP-9) within the lesion was reduced upon MSC transplantation, suggesting that MSCs may protect the ECM and further stabilize the lesion (86). These studies do indicate that MSC treatment can reduce plaque vulnerability by decreasing the regional collagen degradation by inhibiting local protease activity. The apoptosis of vascular ECs, SMCs, and macrophages also contribute to formation, development, and rupture of AS plaques. MSC treatment reduced apoptotic cells within the plaque region, suggesting that MSCs may increase plaque stability (87).

Current strategy of using MSCs as a therapeutic option for AS treatment involves their use as a post-injury treatment approach, exploring the immunomodulatory properties and plaque stabilization effect of MSCs (79–81). Given the broader spectrum of therapeutic responses, MSC treatment might provide an atheroprotective effect and could change the ECM microenvironment to modulate or mitigate early progression of AS (81). Future studies must address the impact of vessel site-specific and temporal differences in the ECM milieu that contribute to AS disease progression. Recent studies have revealed that PGs and pendant GAGs orchestrate AS progression by initiating the binding and retention of atherogenic lipoproteins in the artery intima, leading to foam cell formation (35). Studying the impact of SC treatment in PG remodeling by degrading enzymes at different stages of AS might be interesting and could be used as an early intervention strategy. Likewise, elastin degradation peptides, elastokinaes, effects as modulators of macrophage functions during atherogensis (44) and anti-proteolytic effects of MSCs could be beneficial in reducing local proteolytic activity in the plaque region.

### Stem Cell Therapy for Abdominal Aortic Aneurysm Treatment

AAA pathophysiology is driven mainly by the progressive degradation and loss of ECM structures in the aorta wall, a process driven by the chronic inflammatory milieu in the tissue (48, 49). Therefore, successful AAA treatment strategy must attenuate inflammation, inhibit proteolytic activity, and provide an active stimulus to tropoelastin synthesis and elastic fiber assembly and crosslinking in the aneurysm wall (10). Almost all elastin regenerative repair strategies explored so far are focused on reversing or attenuating adverse signaling pathways or mitigating MMP overexpression within the AAA wall, with limited or no direct emphasis on addressing either poor elastogenesis and ECM regeneration (Table 3). The poor elastin generation capability of terminally differentiated adult SMCs limits their use in cell therapy (106). In this context, SCs represent a viable alternative that could be useful for regenerative tissue repair. Vascular elastin is primarily synthesized and deposited during fetal and neonatal developmental stages in tissue microenvironments rich in stem/progenitor cells. Hence it is reasonable to hypothesize that SCs or their SMC-like derivatives would retain higher elastin production and matrix assembling capabilities than adult aortic cells or AAA-SMCs. An MSC- based treatment approach has demonstrated some promise in attenuating inflammatory processes and proteolytic activity and to stimulate elastogenesis.

**Table 3:** Animal studies involving use MSCs for treatment of Abdominal Aortic Aneurysm.

**AAA model**	**MSC source**	**Major outcomes with MSC treatment**	**References**
AngII infused ApoE-/-mice	BM-MSC (mice)	Reduced MMP2, TNF-α, IL-6, and MCP-1. Increased elastin expression.	(96)
AngII infused ApoE-/-mice	BM-MSC (mice)	Reduction in aortic diameter. Reduced MMP2, MMP9, IL-1β, IL-6, and MCP-1 levels. Preservation of aortic elastin content, increase in IGF1 and TIMP2.	(97)
PPE C57BL/6 mice	AD-MSC (mice)	Reduced aortic diameter. Less fragmented elastin versus saline controls.	(98)
AngII infused ApoE-/-mice	BM-MSC (mice)	Reduced aortic diameter. Reduced MMP2/9 expression, inhibited infiltration of M1 macrophages and preserved elastin.	(99)
PPE-SD Rat	UC-MSC (human)	Reduced aneurysmal expansion, reduced elastin degradation, inhibited MMPs and TNF-α expression.	(100)
AngII infused ApoE-/- mice	Allogenic and Autologous BM-MSC (mice)	No major difference between allogenic and autologous MSC in reducing chronic inflammation and reduced aortic dilation.	(101)
PPE-SD rat	BM-MSC and BM-SMC (rat)	BM-MSC and BM-SMC downregulated expression of several inflammatory and pro-apoptotic cytokines. Deposition of thick and matured fibers were observed with BM-SMC treatment in the AAA wall; thinner and fragmented fiber	(102)
Cacl_2_ infused rat	AD-MSC (mice)	Reduced MMP2 and MMP9 expression Increased elastin expression	(103)
PPE C57BL/6 mice	AD-MSC (human)	Reduced aortic expression and plelotropic immunomodulatory effects. No aortic engraftment following I.V, paracrine effects following lung engraftment	(104)
Angll infused ApoE^−/−^ mice	BM-MSC (mice)	Regulation of the NF-κB, Smad3, and Akt signaling pathways.	(105)

The anti-inflammatory and pro-matrix regenerative properties of MSCs were evaluated in several investigations, using different AAA animal models. These studies are summarized in Table 4. In a seminal study by Hashizume et al. murine MSCs attenuated enzyme activities of both elastolytic MMP2 and MMP9 and reduced production of inflammatory cytokines (i.e., TNF-α) by murine macrophages (96). These cells also stimulated elastin synthesis by murine aortal SMCs in culture. Further *in vivo* studies on male angiotensin II (Ang II) infused ApoE^−/−^ mice demonstrated that MSC implantation *via* laparotomy was effective in downregulating the expression of MMPs, IL-6, Monocyte Chemoattractant Protein-1 (MCP-1), and TNF-α, and also in upregulating Insulin-like Growth Factor-1 (IGF-1) and TIMP-1. Since laparotomy for localized cell delivery to the AAA wall was invasive, BM-MSCs were delivered *via* the intravenous (I.V.) route into an AngII-induced AAA mouse model (97). In this study, multiple BM-SMC dosing events caused a significant decrease in AAA diameter at the ascending and infrarenal levels, relative to sham controls. Reduction in AAA growth was also associated with decreased macrophage infiltration and suppressed MMP-2 and MMP-9 activity in the AAA tissues, as well as enhanced preservation of elastin content in the AAA wall. BM-MSC treatment also reduced levels of the inflammatory cytokines, IL-1β and MCP-1, and increased expression of IGF-1 and TIMP2 in the AAA wall. In a separate study, Blose et al. showed that periadventitial delivery of Adipose derived MSCs (AD-MSCs) to be effective in slowing AAA progression and in preventing fragmentation of the elastic lamelle in a mouse elastase-perfusion model of the disease (98). Effectiveness of SC therapy to re-establish the mechanical properties of damaged abdominal aorta was attempted using a AAA model. Results from these studies suggest that BM-MSC treatment was effective in stabilizing the geometry of AAAs, improving wall stiffness, and decreasing stress variations in the arterial wall of rat AAA model (99). Wen et al. also showed I.V. administration of Umbilical Cord MSCs (UC-MSCs) was effective in attenuating aneurysmal expansion, reducing elastin degradation and fragmentation, inhibiting MMPs and TNFα expression, and to promote contractile phenotype of SMCs in the AAA wall of elastase-induced rat model (100).

**Table 4:** Animal studies for treatment of vascular disorders using SC-derived exosomes.

**Disease/animal** **model**	**MSC source for exosome isolation/ exosome isolation method**	**Major outcomes with exosome treatment**	**References**
AS/ ApoE-/- mice	BM-MSC (mice)/ UC method	Selective uptake of IV injected MSC-exosomes by macrophages in plaque region, reduced plaque area and induced M2 macrophage polarization through miR-let7/HMGA2/NF-kB pathway.	(107)
AS/ ApoE-/- mice	BM-MSC	Exosomes containing miR-21a-5p promoted M2 polarization of macrophages, reduced plaque area and macrophage infiltration by targeting KLF6 and ERK1/2 signaling pathway.	(108)
AS/ ApoE-/- mice	UC-MSC (human)/UC method	miR-145-rich exosomes downregulated JAM-A, reduce AS plaque *in vivo*.	(109)
AAA caused by AS/ AngII infused ApoE-/- mice	BM-MSC (mice)	Attenuated AA progression decreased expression of IL-1β, TNF-α, and MCP-1, and expression of IGF-1 and TIMP-2 increased. Also induced M2 macrophage phenotype and suppressed elastic lamella destruction.	(110)
AAA/ Elastase induced mice model	UC-MSC (human)/UC method	Reduction in aortic diameter, reduced expression of pro-inflammatory cytokines, increase in α SMC expression and decreased elastic fiber disruption.	(111)

A study by Davis et al. showed BM-MSCs derived from female mice to be more effective in attenuating AAA growth in elastase perfused AAA mice model, compared to MSCs derived from male mice, suggesting sex-related differences in SC characteristics and behavior (101). However, this observation has been very inadequately studied and requires further in-depth assessment. Akita et al. on the other hand compared the therapeutic effects of allogeneic and autologous MSC on Ang II- ApoE^−/−^ model of AAAs as an attempt to create an “off-the-shelf” product as required in clinical practice (112). They observed that both allogeneic and autologous MSCs had comparable effect in terms of reducing chronic inflammation and aortic dilatation.

**Figure 4:**
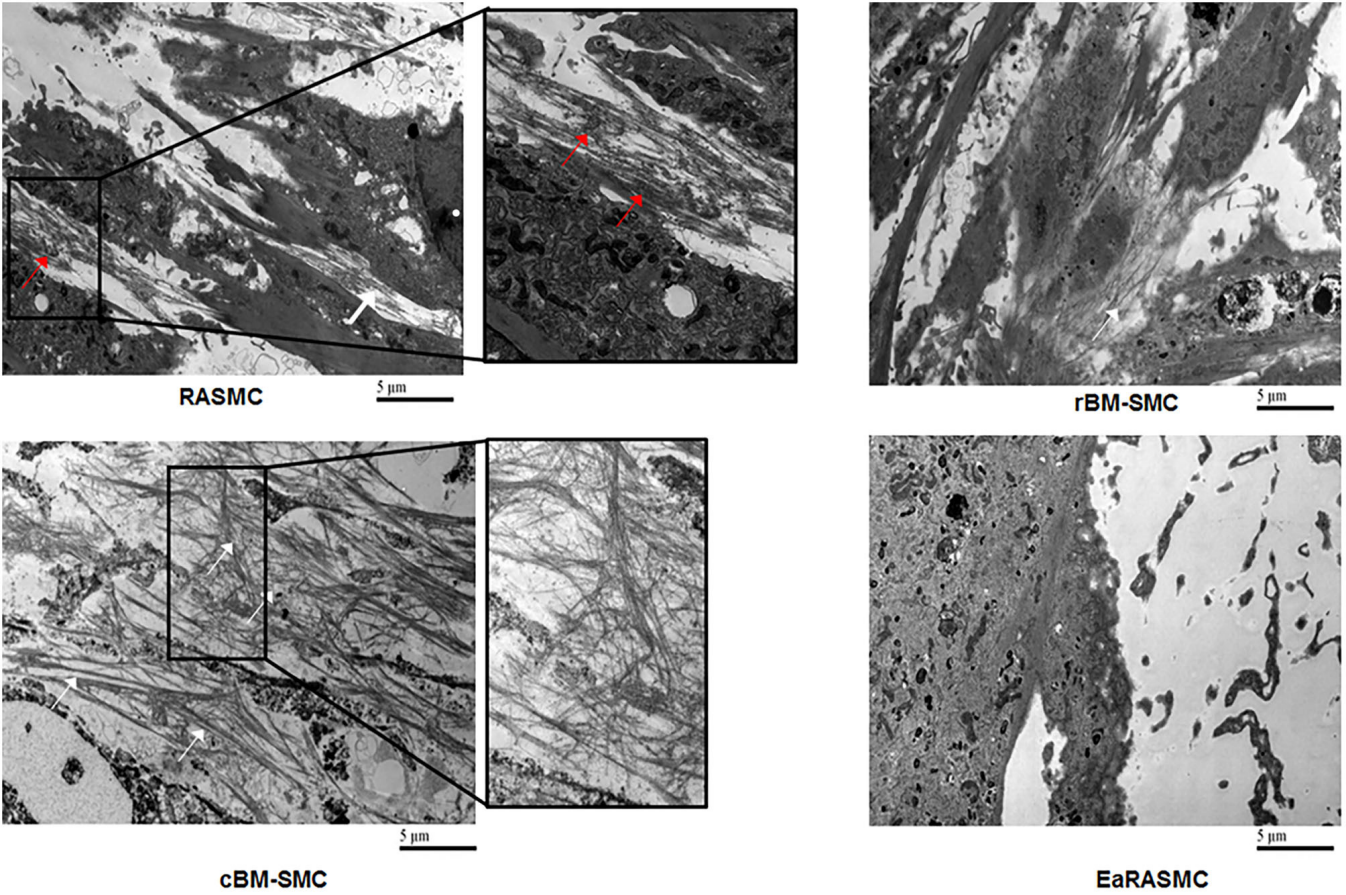
Transmission electron micrographs showing significantly greater density of forming elastic fibers in cBM-SMC cultures, and less so in rBM-SMC cultures relative to RASMC cultures. The elastic fibers were composed of fibrillin microfibrils (white arrows) laid down as a prescaffold onto which amorphous elastin (red arrows) was deposited and crosslinked. The RASMC cultures contained mainly amorphous elastin deposits. Very few amorphous elastin deposits and no fiber-like structures were seen in EaRASMC cultures. Reprinted from Dahal et al. (117), with permission from Mary Ann Liebert, Inc.

Despite the promise of SC therapy for AAA treatment (113), a comprehensive and systematic characterization of the elastogenic capabilities of SCs and their effects on the various steps and processes involved in the complex process of elastogenesis (i.e., elastin precursor synthesis, precursor recruitment and cross-linking, and fiber assembly and organization into superstructures) by healthy and diseased SMCs is lacking. Our research focuses on developing strategies for restoring ECM homeostasis in matrix-compromised vessels, as in the AAA wall, which involves providing a stimulus for on-site regeneration of mature elastic fibers, a deterrent to proteolytic breakdown of existing fibers, and restoring a healthy SMC phenotype. Our earlier investigations using elastase infusion injury rat AAA model provided evidence that neointimal remodeling within aneurysmal tissue is associated with new elastin deposits (114). However, these structures are nascent, do not exhibit the characteristics of mature elastic fibers, and more so, are transient in their presence. The lack of maturity of these structures was suggested to be related to deficient deposition of the fibrillin micofibrils that serve as vital pre-scaffolds on which tropoelastin coacervates get crosslinked toward forming mature elastic fibers. In separate work, we investigated SMC-like cells (BM-SMC) of a defined phenotype differentiated from BM-MSCs, for their effectiveness in (a) augmenting elastic fiber assembly in cultures of aneurysmal SMCs isolated from our elastase injury rat model, and (b) restoring elastic matrix homeostasis in the AAA wall *in vitro* (115, 116). Our cell culture studies showed that BM-SMCs exhibited superior elastogenicity compared to their parent cell (BM-MSCs; Figure 4) and provided pro-elastogenic and anti-proteolytic stimuli to cytokine injured aneurysmal SMCs in culture (117–119). Further, *in vivo* studies suggested that both BM-MSCs and BM-SMCs downregulated expression of several inflammatory and pro-apoptotic cytokines that are upregulated in the AAA wall in the elastase injury rat AAA model, which contributes to accelerated elastic matrix breakdown and suppression of elastic fiber neoassembly, repair and crosslinking (102). Our results also indicated significant improved deposition of thick and matured elastic fibers in the AAA wall upon treatment with the BM-SMCs, whereas the elastic fibers were thinner and fragmented in the BM-MSC-treated and saline-treated (control) animals with AAAs (102). This study demonstrated the promise of using an BM-MSC-derived cells of an SMC lineage for matrix regenerative cell therapy to reverse the pathophysiology of proteolytic disorders such as AAAs.

Overall, MSC therapy was effective in reducing inflammation and ECM degradation at the AAA wall site, through the secretion of several anti-inflammatory cytokines, protease inhibitors and ECM synthesis stimulators (97–100). MSC treatment was also effective in regressing the growth of already-formed AAAs (97, 98, 102, 113). However, with AAA therapy, it is critical to identify specific strategies and mechanisms that are conducive for the repair and assembly of native vascular elastic matrix with high efficiency and fidelity to the fiber assembly process. Future research should focus on understanding the exact healing mechanism of MSCs under AAA conditions, and further assess their impact on the repair and regeneration of ECM components.

### Stem Cell Therapy for Thoracic Aortic Aneurysm and Dissection Treatment

Although SC therapy could play a significant role in tissue repair associated with TAAD, with their ability to attenuate proteolytic activity and improve elastogencity, few studies have addressed this topic. Shen et al. have demonstrated the abundant presence of SCs in the TAAD tissues obtained from patients with descending thoracic aortic aneurysms and with chronic descending thoracic aortic dissections (120). They also observed the differentiation of SCs into SMCs, fibroblasts, and inflammatory cells within the diseased aortic wall and this might suggest the reparative and destructive role of SCs in TAADs. MSC therapy was used to immunomodulate vascular inflammation and remodeling through altering microRNA (miRNAs) expression profile to attenuate TAA formation (121). Descending TAA was induced by topical elastase application in C57BL/6 mice and MSC treatment was effective in attenuating T-cell, neutrophil and macrophage infiltration and prevented elastic degradation to mitigate vascular remodeling. This study also demonstrated miRNA modulating ability of MSCs that are linked to reduce leukocyte infiltration and vascular inflammation to mitigate the aortic diameter and TAA formation in mice. Even though the study outcome was promising, further in-depth investigations are required to evaluate the anti-proteolytic and pro-regenerative benefits of SCs under TAA conditions.

## Stem Cell-Inspired Approaches for Treatment of Common Vascular Disorders

It was originally assumed that SCs regenerate/repair the damaged/diseased regions by homing to the respective locations, engrafting, and subsequently differentiating into mature, functional cells (76, 77). However, this classical hypothesis was confronted by outcomes from studies indicating that SCs are neither engrafted adequately nor retained long enough to explain the tissue replacement/repair process (122). According to a more recent hypothesis, SCs largely employs alternate modes of tissue repair through secretion of paracrine signaling factors, such as cytokines and chemokines, hormones, and extracellular vesicles (EVs) (123–125). These secreted factors, collectively referred as secretome, can be found in the SC culture medium usually mentioned as conditioned media (CM) (126). SC derived CM has been demonstrated to exert several therapeutic benefits by modulating the local immune responses to inhibit inflammation, stimulating ECM remodeling, and decrease cell apoptosis and fibrosis (126). Exosomes seems to be a critical CM component and exosomes derived from therapeutically relevant SC source represent biological functions like the parent cells, by facilitating tissue regeneration/repair through transferring active biomolecules such as peptides, proteins and RNAs to the damaged cells/tissues (122, 123).

## Stem Cell Derived Exosomes for Vascular Tissue Repair

Exosomes, specifically ones derived from MSC sources, have enormous benefits in a variety of diseases and injuries through the secretion of proteins and RNAs that they contain (127). Specifically, MSC-derived exosomes have been investigated for their potential for vascular regeneration and repair, and for treating diseases such as ASs, AAs, stroke, pulmonary hypertension, and septic cardiomyopathy (128–131).

### Biogenesis and Secretion of Exosomes

The term extracellular vesicles (EVs) are used to broadly describe distinct sub-types of extracellular secretions comprised of small bilayer lipid membrane vesicles (132–134). EVs can be classified into three major sub-types based on their mechanism of biogenesis, and size (135). Exosomes, typically in the range of 40–150 nm diameter, are vesicles derived from an endosomal origin and released into the extracellular space following fusion of multivesicular bodies with the plasma membrane. Microvesicles, which are larger in size compared to exosomes (100–1,000 nm diameter), are vesicles that bud out directly into the extracellular space from the plasma membrane without fusion with multivesicular bodies (135). Exosomes and microvesicles are active vehicles for inter-cellular communications, as they are released and taken up by living cells. On the other hand, apoptotic bodies (>800 nm diameter) arise directly from the outward blebbing of the plasma membrane of cells undergoing apoptosis. These particles do not typically participate in cellular communication process (135). Although size is often used to generically classify these sub-types of EVs, exosomes are considered to exhibit diameters of >100 nm and microvesicles to exhibit diameters <100 nm. However, there is a lack of consensus on a strict size cut-off for classifying EVs (136). Several studies have indicated the role of exosome-mediated intercellular communications in maintaining the homeostasis of CV systems, and SC derived exosomes have emerged as an important disease diagnosis/prognosis marker for CV disease and also as a regenerative tool (128, 130).

### Stem Cell Derived Exosomes for Treating Arterial Stiffness and Hypertension

In a study by Feng et al. EVs (exosomes) obtained from iPSC-MSC were used as treatment option for aging-associated arterial stiffness and hypertension (137). I.V. administration of EVs significantly attenuated aging-related arterial stiffness and hypertension, and enhanced endothelium-dependent vascular relaxation and arterial compliance in old male C57BL/6 mice. EV treatment also prevented elastin degradation and collagen I deposition (fibrosis) in older mice and promoted expression of sirtuin type 1 (SIRT1), and endothelial nitric oxide synthase (eNOS) protein expression in aortas. Substantiating this observation, Monroe et al. has reported the capability of MSC-EVs in ameliorating pathological vascular ECM changes in congenital diaphragmatic hernia (CDH)-associated pulmonary hypertension in pregnant rats (138). These studies provide a strong rationale for studying therapeutic potential of SC derived EVs for aging-related vascular diseases and potentially opens new prospect for a non-pharmacological intervention strategy.

### Stem Cell Derived Exosomes for Treating Atherosclerosis

SC-derived exosomes can regulate the incidence and progression of AS and could overcome the limitations associated with conventional AS treatment strategies. MSC-derived exosomes are reported to have an anti-atherosclerotic role (139), whereas exosomes derived from non-stem cell sources, such as neutrophils, macrophages, ECs or vascular SMCs, have a multifaceted role (140, 141).

Studies with AS experimental models clearly suggest that exosomes derived from the MSC source had prominent anti-atherosclerotic effect (107–109, 142) (Table 4). These studies largely focus on the immunomodulatory effect of MSC derived exosome on AS models. MSC-exosomes treatment decreased the AS plaque area in ApoE^−/−^ mice model and greatly reduced the infiltration of macrophages into the plaques, suggesting their anti-atherosclerotic effect. Exosomes were also effective in inducing macrophage polarization toward M2 phenotype *via* up-regulation of miR-let7 (107). Exosomal miRNAs seems to play a vital role in exerting the therapeutic outcomes on AS models (142). Ma et. al. showed that MSC-derived exosomes containing miR-21a-5p promoted M2 polarization of macrophages, and reduced plaque area and macrophage infiltration by targeting KLF6 and ERK1/2 signaling pathways, in an AS model of ApoE ^−/−^ mice fed on a high-fat diet (108). On the other hand, treatment with miR-145-rich MSC-exosomes downregulated expression of Junction Adhesion Molecule A (JAM-A, also known as F11R overexpressed in patients with AS) in human umbilical vein endothelial cells (HUVECs) and further reduced AS plaque area *in vivo* on AS model of ApoE ^−/−^ female mice fed on high-fat diet (109). AD-MSC-derived exosomes restrained the expression of miR-324-5p in a HUVEC lesion model, and this is expected to protect ECs against AS progression (143). miR-100-5 mimic-transfected UC-MSC derived exosomes inhibited inflammatory response in eosinophils *via* a FZD5/Wnt/β-catenin pathway and alleviated AS progression in an ApoE ^−/−^ mouse model (144).

Effect of AD-MSC derived CM in ameliorating AS in Ldlr^−/−^ mice was evaluated and results from this study demonstrated that I.V. injection of MSC-CM suppressed expression of cell adhesion molecules (CAMs) and reduced AS plaque area (87). EVs isolated from these CM also demonstrated immunomodulatory effect under *in vitro* experimental conditions, suggesting the prominent role of EVs in the cell secretome (104). MSC-CM treatment has also shown to inhibit VSMC calcification through the blockade of the bone morphogenetic protein-2 (BMP2)-Smad1/5/8 signaling pathway (145). From these studies it can be assumed that MSC-derived secretomes have prominent anti-atherosclerotic role, however, further in-depth investigations are required to confirm this observation. In addition, role of MSC secreted factors in ECM regulation under AS conditions remains unknown and needs further assessment.

### Stem Cell Derived Exosomes for Treating Aortic Aneurysm

MSC-derived exosomes were evaluated as a therapeutic tool for the mitigation of aortic inflammation and vascular remodeling during AAA formation, as summarized in Table 4. Macrophage derived exosomes are involved in the pathogenesis of AAAs by increasing the MMP-2 expression in VSMC *via* JNK and p38 pathways (146). However, limited studies performed on AAA animal models have demonstrated the pro-regenerative and anti-proteolytic effects of MSC derived exosomes. The therapeutic effects of MSC-exosomes on AAA formation caused by AS were evaluated in an Ang II-infused ApoE ^−/−^ mouse model. The MSC exosomes significantly attenuated AAA progression, reduced expression of pro-inflammatory cytokines and induced M2 phenotype in macrophages (110). The study also confirmed the suppression of elastic lamellae destruction in the aortic wall through MSC-exosome intervention. Spinosa et.al demonstrated that administration of MSC-EVs (exosomes) in an elastase-treated AAA mouse model caused significant attenuation of aortic diameter, reduced expression of proinflammatory cytokines, and decreased elastic fiber disruption, compared with untreated mice (111). The authors further elucidated the role of miR-147 in mediating inflammatory responses in murine aortic tissues treated with elastase. EVs derived from MSCs transfected with a miR-147 mimic attenuated aortic diameter, inflammation, and leukocyte infiltration in elastase-treated mice. Differently, transfection with an miR-147 inhibitor was ineffective in attenuating AAA progression (111).

CM derived from BM-MSC cultures were utilized to treat AAA animal models (AngII-induced AAA in ApoE^−/−^ mice). The results from this study suggest that MSC-CM moderates AAA growth by potentially regulating macrophage polarization and through immunomodulation (147). Use of BM-MSC-CM obtained from male mice failed to attenuate AAA growth in elastae-perfused mice, compared to untreated group, whereas female mice derived BM-MSC-CM was effective in reducing aneurysm growth (101). However, this observation needs further validation.

**Figure 5:**
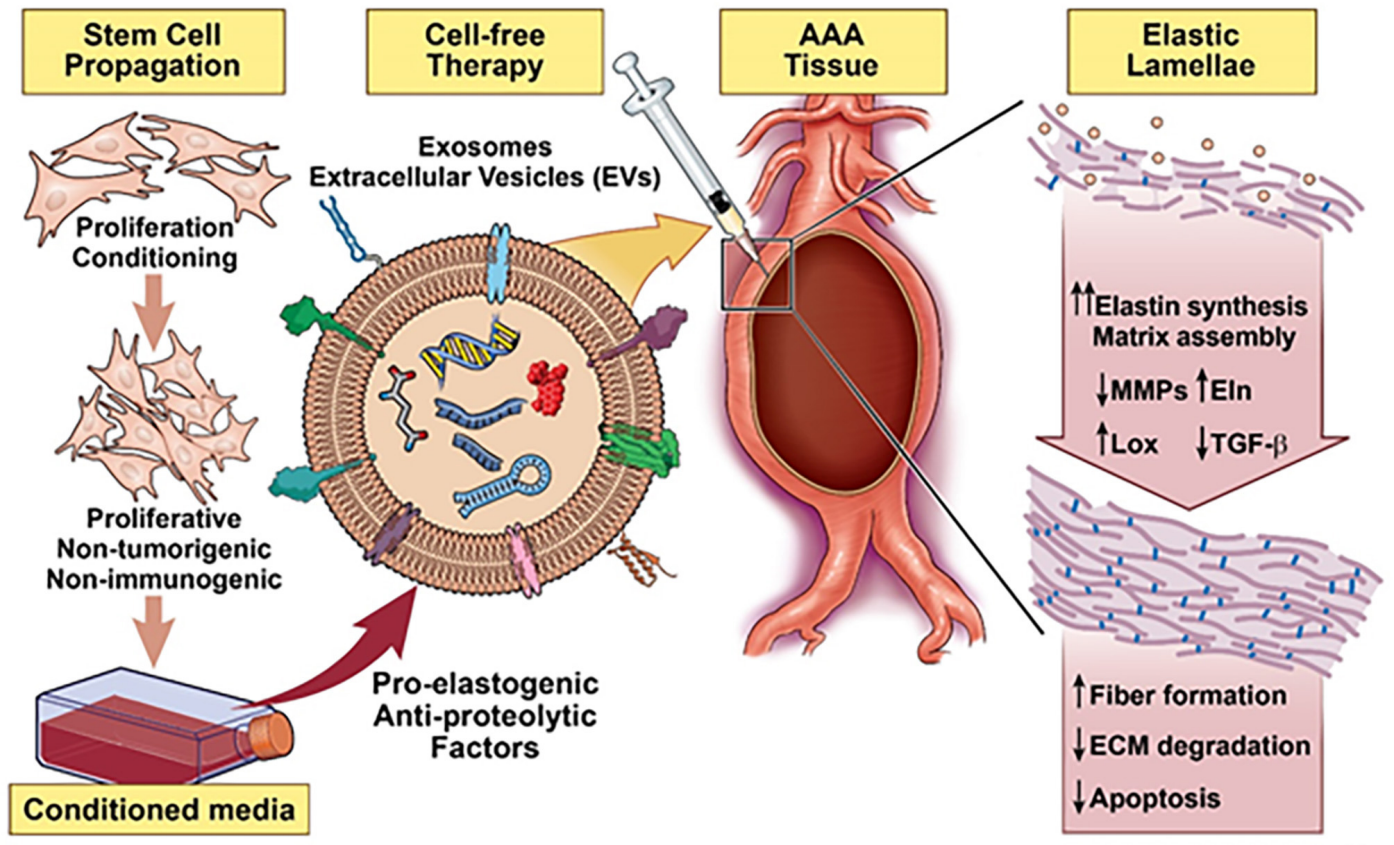
Schematic summarizing a stem cell inspired approach for vascular elastic matrix repair involving delivery of stem cell exosomes.

**Figure 6:**
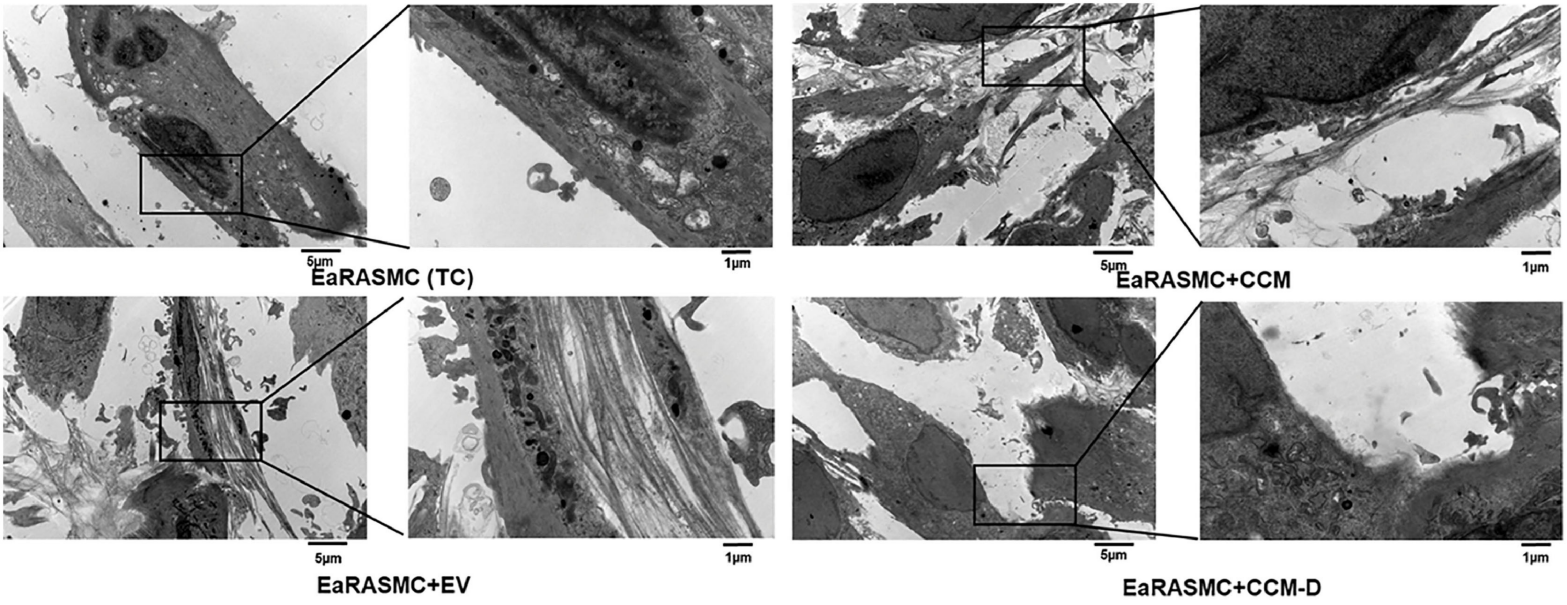
Effects of EV(exosomes)/conditioned media (CCM)/conditioned media depleted with exosome (CCM-D) treatment on elastic fiber ultrastructure. Transmission electron micrographs showing elastic fiber formation (red arrows) in EaRASMC cultures treated with EVs/CCM/CCM-D. TC and CCM-D treated cell layers contained very few sporadic deposits of elastin and no fibers. Reprinted from Sajeesh et al. (148), with permission from Elsevier.

Owing to evidence of the paracrine pro-elastogenic and anti-proteolytic effects of MSC, we explored the regenerative and anti-proteolytic potential of human BM-MSC derived EVs (exosomes) in cytokine-injured cultures of SMCs isolated from the elastase injury induced rat AAAs (148) (Figure 5). Apart from their strong anti-proteolytic effect, the BM-MSC-generated EVs provided effective pro-regenerative cue through the deposition of mature elastic fibers (Figure 6). Additionally, EVs demonstrated superior pro-regenerative and anti-proteolytic effect compared to MSC derived CM and CM depleted with EVs failed to demonstrate any regenerative benefits, in our study. This clearly demonstrates that EVs are necessary component in SC secretions responsible for imparting elastic matrix regenerative and anti-proteolytic properties. Thus, from these limited studies it can be assumed that exosomes recapitulate the therapeutic properties of their parent SCs and may be used as putative surrogate for conventional cell-based therapy.

## Limitations of Using Stem Cell Based Therapy for Vascular Disorders

Despite the significant advances in SC therapy, there are numerous unresolved issues that impede the clinical use of different SCs types including (a) ethical issues concerning use of ESCs, (b) potential tumorigenesis, (c) immune rejection, (d) quality control in sourcing and scale up issues, (e) their uncertain phenotypic state and fate *in vivo*, and (f) challenges to their localized delivery to the site of tissue repair (149). Even with many clinical reports and ongoing clinical trials, the long-term safety and efficacy of SC-based treatments, remain rather vague (150, 151). MSCs are often composed of a heterogeneous mix of different cell population and MSCs from different sources also show variations in their therapeutic efficacy (151, 152). These differences arise largely due to the variation in donors (autologous vs. allogeneic, age, sex, genetics, environmental factors, etc.), different administration routes, dosages, epigenetic reprogramming and senescence followed by culture expansion and cryopreservation (152). Lack of standardized products is a serious impediment for the clinical application of MSCs therapy and limit their therapeutic potential. Systemic administration of MSCs is another major challenge, following reports of MSC entrapment in the microvasculature or lung, usually referred as pulmonary first- pass effect, causing deleterious consequences (153). To overcome these limitations, implementation of good quality control systems is required, and more standardized protocols are mandatory for cell culture and their differentiation, expansion, and cryopreservation.

Efforts should be also made to capitalize the paracrine signaling pathways, as an effort to develop a cell-free approach for regenerative therapeutics. While exosomes promise unparalleled advantages over cell-based therapy (122), their future clinical translation is contingent on overcoming several critical impediments. Lack of standardized method for the collection, isolation, and analysis of exosomes is a significant barrier for the comparability and reproducibility of the results (154). Their variable composition and presence of large number of bioactive agents might induce undesirable effects (155). Site specific delivery of exosomes remains another major challenge to tackle. I.V. administrated exosomes usually have short half-life in the body and are quickly cleared by immune cells and specific strategies must be devised to home exosomes to the pathological sites (156). Implementation of a standardized approach is required for isolation, purification and analysis of exosomes, and precise understanding of their interaction with damaged tissues is required for their potential application.

Another limitation in studying in chronic immunometabolic vascular diseases is the fact that most of these diseases evolve in humans over a period through the involvement of several organs and immune cell type (157). Animal models frequently used to study these diseases are generated in a short time frame and have variations in their metabolism and other inflammatory responses. Most of these vascular diseases are multifactorial in nature and many physiological processes contribute for their progression. Models generated of small laboratory animals (rats, mice etc.) might provide some valuable insights (158, 159), however these models have certain limitations in assessing the exact immunomodulatory responses imparted by SC therapy and their long-term implications. Additionally, most of these studies have not evaluated the impact of SC treatment in ameliorating pathological ECM changes associated with vascular disorders, which is critical toward the re-instatement normal vascular hemostasis. Future studies should aim to identify and establish new triggers and mechanisms involvement in vascular disease development and be more adaptive for evaluating novel treatment approach that accelerate translational cardiovascular research.

## Summary and Future Outlook

Changes in the vascular ECM microenvironment is pervasive across a wide spectrum of vascular disorders. In many instances, these changes have been shown to drive the progressive pathophysiology of these disorders through signaling feedback. Our comprehensive understanding of the dynamic interplay between altered ECM state and dysregulation of vascular cell signaling provides new insight that might guide toward development of new treatment approaches to either prevent or to actively regress these vascular pathophysiologies.

Recent advancement in SC technologies have brought MSCs to progress closer to clinical applications for disease therapy and tissue reconstruction, even though challenges might seem daunting. The immunomodulatory effect of SCs at vascular injury sites, followed by ECM regenerative effect induced *via* secretion of growth factors have demonstrated efficacy not only in prevention of diseases but also in the regression of damaged vascular tissues. This synergistic immunoregulatory effect combined with matrix regenerative abilities of SC-based and inspired products may help in developing novel therapeutic strategies for preventing and treating vascular disorders. The clinical translation of these therapeutic strategies is however conditional on progress in addressing key challenges associated with the clinical translation of SC technology.

## Author Contributions

SS and AR conceptualized and wrote the manuscript. SDah, SDay, SB, and JY helped in the collection of literature and critically discussed the content. All authors reviewed the manuscript before submission and have read and agreed to the publication of the manuscript.

## Funding

The AR lab has been funded by the following agencies in the USA for the following projects: National Institutes of Health (NHLBI), HL 139662-01. Title: Matrix Regenerative Nanotherapeutics for Abdominal Aortic Aneurysm Repair. National Science Foundation, CBET 1926939, Collaborative Research: Design and development of a multifunctional nanoplatform for augmented elastic matrix repair. American Heart Association, 19TPA34890029, Matrix regenerative siRNA nanotherapeutics for small aneurysm repair. Discretionary funds from Lehigh University usable to cover publishing costs.

## Conflict of Interest

The authors declare that the research was conducted in the absence of any commercial or financial relationships that could be construed as a potential conflict of interest.

## Publisher's Note

All claims expressed in this article are solely those of the authors and do not necessarily represent those of their affiliated organizations, or those of the publisher, the editors and the reviewers. Any product that may be evaluated in this article, or claim that may be made by its manufacturer, is not guaranteed or endorsed by the publisher.
